# Salt Taste Genotype, Dietary Habits and Biomarkers of Health: No Associations in an Elderly Cohort

**DOI:** 10.3390/nu12041056

**Published:** 2020-04-10

**Authors:** Celeste Ferraris, Alexandria Turner, Kiranjit Kaur, Jessica Piper, Martin Veysey, Mark Lucock, Emma L. Beckett

**Affiliations:** 1 School of Environmental and Life Sciences, The University of Newcastle, Ourimbah, NSW 2258, Australia; celeste.ferraris@uon.edu.au (C.F.); Alexandria.Turner@uon.edu.au (A.T.); KiranjitKaur1@uon.edu.au (K.K.); Jessica.piper@uon.edu.au (J.P.); mark.lucock@newcastle.edu.au (M.L.); 2 School of Medicine and Public Health, The University of Newcastle, Gosford, NSW 2250, Australia; martin.veysey@hyms.ac.uk; 3 Hull York Medical School, University of Hull, Heslington HU6 7RX, UK; 4 Hunter Medical Research Institute, New Lambton Heights, NSW 2305, Australia; 5 Priority Research Centre for Physical Activity and Nutrition, The University of Newcastle, Callaghan, NSW 2308, Australia

**Keywords:** salt, taste, *TRPV1* gene, rs806500, dietary, biomarker, elderly, nutrigenetics

## Abstract

A small amount of emerging research has observed variations between individual sensitivity, preference and intake of salt in the presence of single nucleotide polymorphisms (SNP) on the genes encoding salt taste receptors. Sodium intake is a significant risk factor for common diseases in elderly populations such as hypertension and cardiovascular disease; however, this does not fully explain the risk. Research into the influence of salt taste genetics on diet quality is yet to be undertaken and current research on indicators of health is limited and mixed in the direction of associations. Therefore, a secondary analysis of data from a well-characterised elderly cohort (the cross-sectional Retirement Health and Lifestyle Study, *n* = 536) was conducted to explore relationships between the salt taste-related SNP *TRPV1*-rs8065080 (assessed by Taqman genotyping assay), dietary habits and biomarkers of health. Data were analysed with standard least squares regression modelling and Tukey’s HSD post hoc tests. No association was found between the *TRPV1*-rs8065080 genotype, sodium intake or multiple diet quality indices (assessed by food frequency questionnaire). Sodium-related markers of health including blood pressure and markers of kidney function (urinary creatinine and albumin/creatinine ratio) and general health markers, such as Body Mass Index (BMI), were also not related to *TRPV1*-rs8065080 genotype. To date, this study is the most comprehensive investigation conducted to determine if the *TRPV1*-rs8065080 genotype relates to sodium intake and health markers influenced by sodium intake. Although no significant relationships were found, these findings are an important contribution to the limited body of knowledge surround this SNP. In addition to further research across other ages and cultures, the *TRPV1*-rs8065080 genotype may interact with other ion channels, and so further studies are required to determine if polymorphic variations influence sodium intake, diet and health.

## 1. Introduction

With an ageing population [1] and diet known to be a major modifiable determinant of disease risk, understanding the relationships between taste status, diet and health status may be important in detecting and managing at-risk groups [2]. Excess sodium intake in the elderly increases the risk of cardiovascular disease, hypertension [3], osteoporosis [4] and gastric cancers [5]. However, in most countries, salt intake levels remain higher than the World Health Organizations’ recommendations [6]. Furthermore, sensitivity, preference and intake of salt vary widely between individuals. The influence of genetics on these factors has been demonstrated across each of the five tastes, including salt in limited early research [7,8]. Individual differences in salt taste perception have been attributed to variations on the genes encoding taste receptors [9,10]. Evidence for the role of genetics in salt taste and health is emerging, and therefore, many relationships remain to be characterised.

Salt taste is detected through ion channels [11]. Along with the epithelial sodium channel (ENaC), the transient receptor potential cation subfamily V member 1 (TRPV1) channel has been identified as a salt taste receptor that responds to a variety of cations [12]. The TRPV1 gene is located on chromosome 17 [13] and is expressed throughout the body [14]. Salt concentration levels elicit different taste pathways. Type 1 taste cells have been identified as activated when concentrations are appetitive [15], while Type II and III taste cells are triggered by higher salt concentrations that are perceived as aversive [15]. Furthermore, single nucleotide polymorphisms (SNP’s) on the TRPV1 gene have been identified as having influence on the threshold levels at which individuals perceive salt solutions [9].

*TRPV1*-rs8065080 is a missense mutation with the single amino acid change from isoleucine to valine occurring at position 585 [13]. The frequency of alleles differs between populations. The *TRPV1*-rs8065080 T allele is more common in Caucasian, African and Hispanic populations, and the C allele more common in Asian populations [13]. Animal and cell culture studies demonstrate potential mechanistic roles for the *TRPV1*-rs8065080 polymorphism in ion channel function. Transfection of HeLa cells with the *TRPV1*-rs8065080 C allele results in a 20%–30% loss of channel function [16]. Furthermore, salt-sensitive rats on a high salt diet had reduced expression and function, with authors hypothesizing that this was a potential mechanism for salt sensitivity [17].

Human research on the function and dietary outcomes of the different *TRPV1*-rs8065080 variants is limited and mixed. In a study of ten TRPV1 SNPs (*n* = 95, white, young adults), *TRPV1*-rs8065080 was the only SNP identified as being related to salt suprathreshold taste sensitivity [9]; however, liking and salt intake were not assessed. In a sub-study of participants from the Guelph Family Study (*n* = 125) various TRPV1 SNPs, but not *TRPV1*-rs8065080, were found to be associated with higher sensitivity to, and preference for, salt [18]. Sodium intake was assessed by three TRPV1 SNP’s (rs4790151, rs4790522 and rs877610); however, no significant relationships were found [18]. A smaller American study (*n* = 20), in which the *TRPV1*-rs8065080 T allele was associated with higher sensitivity to salt, found T allele carriers consumed higher amounts of sodium than C allele carriers [10]. However, the small cohort size in the study limits the power of the analysis. In addition to salt taste, a large Korean epidemiology study (*n* = 8842) found *TRPV1*-rs8065080 T allele carriers to have a higher preference for, and consumption of oily foods [19]. The varied relationships demonstrate SNP-related functional impacts on taste that require further definition.

The health outcomes of *TRPV1*-rs8065080 variants have been investigated in both sodium-related and non-sodium-related diseases. While sodium intake is an established risk factor in high blood pressure [3], a recent Taiwanese study did not find a connection to the TRPV1 SNP [20]. In male and female adults (*n* = 551), *TRPV1*-rs8065080 was not associated with systolic or diastolic blood pressure levels [20]. Conversely, the *TRPV1*-rs8065080 allele carriage has been associated with risk for other diseases not directly related to sodium intake, including type 2 diabetes and insulin sensitivity [19], the risk for knee osteoarthritis [21], cough and wheeze in asthmatics [16,22], and differential responses to pain [23,24].

The relationships between the *TRPV1*-rs8065080 polymorphism, salt intake and markers of health related to sodium intake remain to be fully elucidated. Therefore, we assessed the relationship between the *TRPV1*-rs8065080 genotype, sodium intake, diet quality, Body Mass Index (BMI), blood pressure and markers of kidney function in a well-characterised elderly cohort.

## 2. Materials and Methods

### 2.1. Subjects

This secondary cross-sectional analysis examined data from the Retirement Health and Lifestyle Study (RHLS) conducted on the NSW Central Coast of Australia from 2010 to 2012 [25]. Individuals living in private dwellings were randomly selected from extracts of the Wyong and Gosford local government areas Australian Commonwealth Electoral Rolls. Individuals from 12 participating retirement villages located in the same electorates were also randomly selected from retirement village resident lists. Participants were eligible to participate if they were aged 65 years or older and their primary residence for the last 12 months or more was located within the Wyong or Gosford local government areas. Those who were not living independently or were residing in a communal setting other than a retirement village, had been living in the area for less than 12 months, and/or were in the process of relocating, were not eligible to participate. Individuals were also ineligible if another member of their household was taking part in the study. People with language or other communication difficulties, who were cognitively impaired or unable to provide informed consent, were also excluded [26]. Participants were not excluded based on pre-existing health conditions. In total, 831 people were recruited for this study, however, the provision of a blood sample for genotyping was optional. Only those who were successfully genotyped for *TRPV1*-rs8065080 and provided a valid food frequency questionnaire were included in this sub-study. Complete data sets were available for 536 participants. Ethics approval for the RHLS study was granted by The Human Research Ethics Committee of the University of Newcastle (Reference No. H-2008-0431) and written consent obtained from participants [27].

### 2.2. Anthropometric Measures

Age, sex, education and income levels were collected via questionnaires. Anthropometric measures followed the International Society for the Advancement of Kinanthropometry (ISAK) guidelines [28]. Participants wore light comfortable clothing and measures were repeated until two consecutive values within 0.5 cm were recorded. The stretch stature method was used to measure height and recorded to the nearest 0.01 cm [28]. Digital scales (Wedderbum^©^ UWPM150 Platform Scale) measured weight which was recorded to the nearest 0.01 kg. Calculations using the height and weight measures determined Body Mass Index for each participant (BMI = weight (kg)/height (m^2^)). Waist, hip and waist to hip ratio measures were also taken, following the ISAK guidelines [28].

### 2.3. Blood Pressure Readings

Blood pressure (BP) measurements were administered by qualified clinical staff using an OMRON IA2 machine. Readings were taken from both arms, allowing at least one minute between measurements. In the arm with the highest reading, a further two BP measurements were taken and recorded, these two consecutive measurements were averaged and used in statistical analyses. If the consecutive readings differed by >10 mmHg for systolic blood pressure or by >6 mmHg for diastolic blood pressure, a further fourth measurement was taken. Measurement was abandoned after a maximum of four readings (on one arm). Participants were excluded if their measurement was invalid (consecutive BP readings differed by more than 10 mmHg systolic or 6 mmHg diastolic); there was a physical limitation preventing measurement; they presented with a very high BP curtailing measurement, or there was a machine error.

### 2.4. Collection of Biological Samples

After fasting, whole blood was collected via venipuncture, by a trained nurse, into EDTA-lined tubes and stored at −20 °C [27]. Urine samples were also collected without preservative on the morning of the clinic visit while the participant was fasting. Urinalysis was conducted by the Hunter Area Pathology Service.

### 2.5. Genotyping

In the RHLS study, deoxyribonucleic acid (DNA) was isolated from peripheral blood cells with the QIAGEN QIAmp DNA mini-kit following the manufacturers’ protocol [25,29]. The DNA samples were stored at −20 °C [25]. The SNP was assessed with quantitative polymerase chain reaction (qPCR) in the QuantStudio 7 Flex Real-Time PCR System [30]. Allelic discrimination for *TRPV1*-rs8065080 was performed using TaqMan™ assay code C___11679656_10 (Applied Biosystems™, ThermoFisher Scientific, CA, USA). In 384 well plates (MicroAmp^®^ Optical 384-Well Reaction Plate with Barcode, Applied Biosystems™, ThermoFisher Scientific, CA, USA), 2.25 µL of DNA was dried down in each well. Each qPCR reaction contained the DNA and 2.50 µL 2x TaqMan™ Master Mix, 0.25 µL 20x TaqMan™ Assay and 2.25 µL nuclease-free water (UltraPure™ Distilled Water, Invitrogen, ThermoFisher Scientific, CA, USA). Two no-template controls with all components except DNA were run for specificity validation. The plates were sealed (MicroAmp^®^ Optical Adhesive Film, Applied Biosystems™, ThermoFisher Scientific, CA, USA) and centrifuged (SelectSpin™ Plate Centrifuge, Select BioProducts, NJ, USA). Denaturing occurred for 10 min at 95 °C initially, then 15 s at 95 °C over 40 cycles. Elongation and annealing ran for 10 min at 60 °C. Data were captured at the end of each cycle.

### 2.6. Dietary Assessment

A food frequency questionnaire (FFQ), containing 225 items, and covering all food groups [31] was used to estimate daily sodium intake and to calculate the diet quality indices. Data were analysed using Foodworks 2.10.146 (Xyris Software, Brisbane, QLD, Australia) [32]. Three diet quality indices were calculated from the available data: The Dietary Guideline Index (DGI) [33], the Australian Recommended Food Score (ARFS) [34,35] and the Australian Healthy Eating Index (Aust-HEI) [36].

The DGI is a 150-point index based on the Dietary Guidelines for Australian Adults, Australian Guide to Healthy Eating, national indicators for food and nutrition and the Australian Alcohol Guidelines [33,37,38]. A score of 0–10, proportionally adjusted, was allocated across 15 food categories, with higher scores indicating higher quality dietary intake [33]. The fifteen constituents of the DGI are set to assess a participant’s intake of key nutrients from core food groups, the proportion of key nutrient intakes from healthy food types (e.g., lean meats or wholegrain cereals), diversity of foods in the diet and intakes of unhealthy foods.

The ARFS reflects the Australian Dietary Guidelines and focuses on dietary variety as an indicator of diet quality [34,35]. Points are allocated within eight food sub-scales (vegetables, fruits, protein sources, grains, dairy, fats and alcohol). Points are assigned based on of frequency of consumption reflective of the guidelines. The ARFS score was calculated by summing the points for each item. A maximum of 74 points were possible, with higher scores indicating a wide variety in dietary intakes [35]. Minor modifications were made to the ARFS scorings system due to differences in the foods listed on the FFQ used. Garlic, beetroot and zucchini were not listed on the FFQ used here, but cucumber, asparagus and sweet potato were and so were substituted in the vegetable sub-scale. Similarly, frozen or canned fruits and pears were substituted for berries and kiwifruit/plums/grapes in the fruit sub-scale. In the grains sub-scale, the all-bran was replaced with all high fibre cereals not already captured. In the protein foods sub-scale, veal was not listed in the FFQ, but turkey/quail/duck were and so they were substituted. Alcohol consumption was recorded as an average so consumption per sitting could not be calculated. Points were awarded based on average annual consumption equivalent to 1 or 2 drinks per day for a maximum of four days of the week. Zero points were awarded to those who consumed more than this or never consumed alcohol.

The Aust-HEI generates a score for healthy dietary behaviours and food consumption, equally weighting dietary variety, measures of healthy choices, fruit and vegetable consumption, fat consumption and consumption of discretionary foods [36]. Possible scores range from 0–60 points.

### 2.7. Medical History and Medication Status

Self-reported medical history of cardiovascular diseases (hypertension, heart disease, stroke, heart attack and vascular disease) and kidney disease were recorded via interviewer administered questionnaire. Regular prescription medication use was recorded via presentation of medication packages to investigators of photographic recording of brand, active ingredient and dose, and self-report of frequency of use via interviewer administered surveys.

### 2.8. Statistical Analysis

Data analyses were completed with statistical analysis software JMP (Version 14.2; SAS Institute Inc., USA). Participant characteristics were reported as number and percentage of the total cohort when variables were categorical and mean, minimum and maximum ranges and standard deviation (SD) when continuous. Genotype allele frequency was reported as number and percentage with distributions analyzed using Pearson’s chi-squared tests. Associations between genotype and the continuous variables were assessed with standard least squares regression. Least squared means were compared with appropriate adjustments, or raw means unadjusted, using Tukey’s HSD post hoc test. The threshold for statistical significance was *p* = <0.05. Stratified analyses by each of the potential confounding variables are also presented where appropriate.

## 3. Results

### 3.1. Participant Characteristics

After exclusions, data for 536 participants were available for analysis. Participant age ranged from 65 to 94 years (mean 77.4 years, standard deviation of 6.8 years, Table 1). The mean BMI of participants was in the overweight range (28.5; Table 1). The mean DGI was 97/150, the mean ARFS was 26.8/74 and the mean AUST-HEI was 30.2/60. Mean estimated sodium intake was 2052 mg/day.

All dietary indices and BMI were normally distributed. 45% of participants were male. The majority reported incomes in the middle-income bracket with education levels of TAFE qualification or higher (Table 2). Due to the small numbers of participants in the higher income bracket, this variable was collapsed into two groups for analysis (above AUD 20,000 and below AUD 20,000). Similarly, ex-smokers and current smokers were collapsed into “ever” smokers for analysis.

### 3.2. Genotype Distributions

The variant *TRPV1*-rs8065080 allele (C) had a frequency of 0.36, and the ancestral allele (T) had a frequency of 0.64. The heterozygous genotype (C/T) was the most common, followed by T/T and C/C (Table 3). The distributions of age (*p* = 0.5), sex (*p* = 0.7), income (*p* = 0.5), education (*p* = 0.6), history of cardiovascular disease (*p* = 0.2), history of kidney disease (*p* = 0.5), use of antihypertensive medication (*p* = 0.1) and use of any prescription medication (*p* = 0.1) did not vary significantly by *TRPV1*-rs8065080 genotype.

### 3.3. Sodium Intake by Genotype

**Figure 1:**
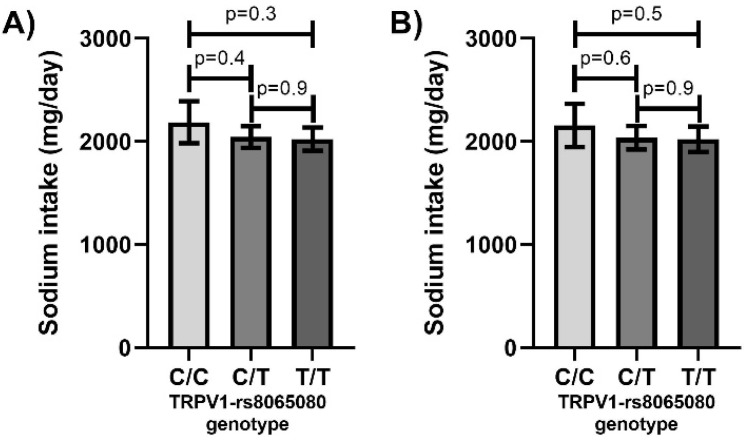
Sodium intake does not vary by TRPV1-rs8065080 genotype. (**A**) Unadjusted mean values (**B**) Least-squares means with adjustments for age, income and sex. Error bars mark 95% confidence intervals.

As TRPV1 is involved in detecting salt taste, the relationship between sodium intake and *TRPV1*-rs8065080 genotype was assessed. Sodium intake was higher in males than females with mean intakes of 2226 ± SD814 mg/day and 1910 ± SD840 (*p* < 0.001) and was higher in the higher income group (1906 ± SD924 mg/day vs. 2129 ± SD8044 mg/day, *p* = 0.005). Intake also reduced with age (β = −0.13, *p* = 0.004). However, sodium intake was not related to smoking status (*p* = 0.9), education (*p* = 0.9), history of cardiovascular disease (*p* = 0.9, history of kidney disease (*p* = 0.3), use of anti-hypertensive medication (*p* = 0.5) or use of any prescription medication (*p* = 0.6). Therefore, sodium intake was assessed by genotype in the complete cohort without adjustments, and with adjustments for age, income and sex. However, there were no significant differences in sodium intake by genotype (Figure 1). The association remained non-significant when salt intake was categorically analysed by quartiles (χ^2^ =4.8, *p* = 0.3). Furthermore, there were no significant differences in salt intake by genotype when analyses were stratified by each of the potential adjustment variables (Table S1).

### 3.4. Diet Quality by Genotype

**Figure 2:**
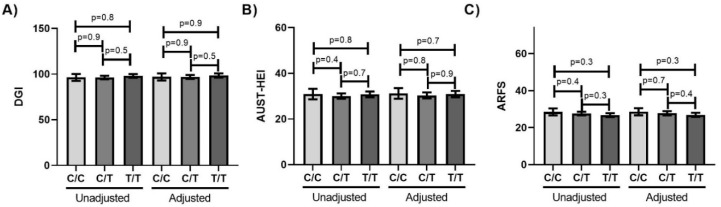
Diet quality indices do not vary by TRPV1-rs8065080 genotype. (**A**) DGI (**B**) AUST-HEI (**C**) ARFS. Error bars mark 95% confidence intervals. Unadjusted present mean values with no adjustments. Adjusted present Least-squares means with adjustments for sex, education and smoking.

It is often hypothesized that taste genetics are involved in modulating dietary preferences and intake. Therefore, we used three diet quality indices to assess the relationship between *TRPV1*-rs8065080 genotype and diet quality. DGI and AUST-HEI were higher in females than males and both increased with education (Table S2). The DGI was also higher in those who had never smoked, compared to those with a history of smoking (Table S2). ARFS did not vary by sex, education or smoking status (Table S2). None of the diet quality indices varied by income (Table S2), age (DGI *p* = 0.1, AUST-HEI *p* = 0.2, ARFS *p* = 0.2), history of cardiovascular disease (DGI *p* = 0.2, AUST-HEI *p* = 0.2, ARFS *p* = 0.3), history of kidney disease (DGI *p* = 0.4, AUST-HEI *p* = 0.2, ARFS *p* = 0.2), use of anti-hypertensive medication (DGI *p* = 0.3, AUST-HEI *p* = 0.3, ARFS *p* = 0.1) or use of any prescription medication (DGI *p* = 0.2, AUST-HEI *p* = 0.1, ARFS *p* = 0.1). Therefore, the relationship between diet quality and *TRPV1*-rs8065080 genotype was assessed without adjustments, and with adjustments for sex, education and smoking. However, there were no significant differences in any of the diet quality indices by genotype (Figure 2) Furthermore, there were no significant differences in any of the indices by genotype when analyses were stratified by each of the potential adjustment variables (Table S1).

### 3.5. Markers of Health by Genotype

**Figure 3:**
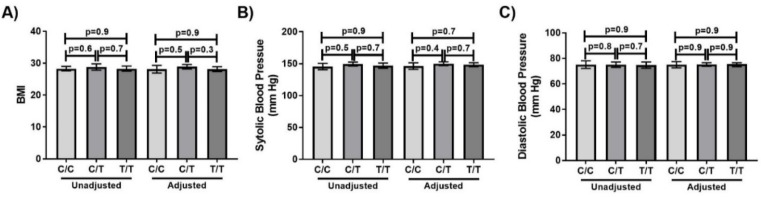
BMI, systolic blood pressure and diastolic blood pressure do not vary by TRPV1-rs8065080 genotype in the elderly. (**A**) BMI (**B**) Systolic blood pressure (**C**) Diastolic blood pressure. Error bars mark 95% confidence intervals. Unadjusted present mean values with no adjustments. Adjusted present Least-squares means with adjustments for age, smoking status, education and income.

The relationship between *TRPV1*-rs8065080 genotype and BMI was assessed as a marker of health status. BMI reduced with age (β = −0.17, *p* = 0.0001), was higher in those who had a history of smoking (29.1 ± 5.1 vs 27.7 ± 5.6, *p* = 0.005), and reduced with increasing education (β = 0.12, *p* = 0.03) and income (β =0.13, 0.01). BMI did not vary by sex (*p* = 0.7) history of cardiovascular disease (*p* = 0.1), history of kidney disease (*p* = 0.4), use of anti-hypertensive medication (*p* = 0.2) or use of any prescription medication (*p* = 0.1). Therefore, the relationship between BMI and *TRPV1*-rs8065080 genotype was assessed without adjustments, and with adjustments for age, smoking status, education and income. However, BMI did not vary by genotype (Figure 3A).

The relationship between *TRPV1*-rs8065080 genotype and blood pressure was assessed as a marker of a salt-sensitive health outcome. Systolic and diastolic blood pressure both increased with age (*p* = 0.003 and *p* = 0.0001, respectively), and were higher in males (*p* = 0.003 and *p* = 0.006, respectively) and those who had a history of smoking (*p* = 0.03 and *p* = 0.02, respectively). Blood pressure did not vary by education (*p* = 0.9), income (*p* = 0.9) history of cardiovascular disease (*p* = 0.2), history of kidney disease (*p* = 0.1), use of anti-hypertensive medication (*p* = 0.2) or use of any prescription medication (*p* = 0.1). Therefore, the relationship between blood pressure and the *TRPV1*-rs8065080 genotype was assessed without adjustments, and with adjustments for age, smoking status, education and income. However, neither systolic nor diastolic blood pressure varied by genotype (Figure 3B,C). The incidence of hypertension did not vary between genotypes (χ = 0.5, *p* = 0.8). Results did not vary when adjustment for sodium intake was added, and there was no significant interaction between *TRPV1*-rs8065080 genotype and sodium intake in predicting systolic or diastolic blood pressure (p_interaction_ = 0.6 and 0.7, respectively). Furthermore, there were no significant differences these markers by genotype when analyses were stratified by each of the potential adjustment variables (Table S1).

The relationship between the *TRPV1*-rs8065080 genotype and urine creatinine and albumin to creatinine ratio was assessed as a marker of kidney health. Urine creatinine was inversely related to age (β = −0.14. *p* = 0.001), was higher in males (8.5 ± SD5.0, v 10.2 ± SD5.2, mmol/L, *p* = 0.0006) and those with a history of kidney disease (8.9 ± SD2.0, v 10.0 ± SD4.2, mmol/L, *p* = 0.02). Urine markers did not vary by education, income, smoking history, history of cardiovascular disease (*p* = 0.9), use of anti-hypertensive medication (*p* = 0.2) or use of any prescription medication (*p* = 0.3). Therefore, the relationship between urine markers and *TRPV1*-rs8065080 genotype was assessed without adjustments, and with adjustments for age and sex. However, neither urine creatinine nor the albumin to creatinine ratio varied by genotype (Table 4). Results did not vary when adjustment for sodium intake was added, and there was no significant interaction between *TRPV1*-rs8065080 genotype and sodium intake in urine creatinine, albumin or albumin to creatinine ratio (p_interaction_ = 0.7 and 0.5, respectively). Furthermore, there were no significant differences in salt intake by genotype when analyses were stratified by each of the potential adjustment variables (Table S1).

## 4. Discussion

This study is the most comprehensive characterisation of the relationships between the *TRPV1*-rs8065080 genotype, sodium intake and health markers influenced by sodium intake to date. Despite the suggested role for *TRPV1* in the detection of salts [12] including sodium [9,10], no association was found between the SNP, sodium intake or sodium-related markers of health including blood pressure and markers of kidney function in the elderly. Additionally, although salt preference potentially influences dietary patterns and quality, BMI and multiple diet quality indices were not related to the *TRPV1*-rs8065080 genotype.

The data presented here, in elderly participants, supports the finding for *TRPV1*-rs4790151, -rs4790522 and -rs877610 and sodium intake in a previous smaller study (*n* = 125) which found that these SNPs were not associated with sodium intake in adults and preschool-aged children. However, it is contradictory to the data presented in a small study (*n* = 20) of young predominantly Caucasian participants by Pilic et al. [10], who reported that the *TRPV1*-rs8065080 T allele carriers consumed higher amounts of sodium than C allele carriers. From an SNP perspective, the *TRPV1*-rs8065080 missense mutation alters one amino acid and, therefore, direct comparison is only possible between this study and the work of Pilic et al. [10]. The contradictory results may be explained by the significant difference in ages between the two cohorts, as age has been found to be a factor in phenotypical variance in genetic expression [39]. However, the limited research available in the genetics of salt taste restricts the valuable comparative analysis, highlighting the need for studies such as this that contribute to the characterisation of TRPV1 SNPs.

Despite no relationship between the presence of the SNP and sodium intake being found, assessing the relationship between *TRPV1*-rs8065080 genotype, blood pressure and kidney function remained important due to the extra-oral expression of TRPV1 throughout the body [14]. Extra-oral taste receptors act as chemosensors [40] and therefore may have biological impacts independent of the modulation of diet via taste thresholds and preferences. However, no association was found between blood pressure and the *TRPV1*-rs8065080 genotype in the present study. This supports the findings of a similarly sized study in male and female Taiwanese adults (*n* = 551) which found no association between *TRPV1*-rs8065080 and systolic or diastolic blood pressure levels [20]. Together, these two well-sized studies suggest that the *TRPV1*-rs8065080 genotype does not directly relate to blood pressure in adulthood nor in later life of the elderly. Demonstrating the presence or absence of this relationship in the elderly is important, as ageing is known to heighten the effect of sodium intake on blood pressure [41]. The present study is the first to assess the relationship between the *TRPV1*-rs8065080 genotype and markers of kidney function; therefore, the absence of association is novel.

The relationship between the *TRPV1*-rs8065080 genotype and broader markers of dietary composition, including BMI and diet quality indices, has not been explored previously. This approach was taken to reflect overall dietary composition, as dietary patterns are important in determining disease risk. Furthermore, a nutrient-focused reductionist approach may miss important and relevant associations. However, the *TRPV1*-rs8065080 genotype was not related to diet quality by any of the indices used and was not related to BMI.

Strengths of this study include the large-sized and well-characterised nature of the cohort that is representative of the larger population. The *TRPV1*-rs8065080 allele frequencies in participants matched those found in global populations [13]. The mean BMI (28.5 kg/m^2^) placed the study population in the overweight category, and this was reflective of the national average for older adults [42]. The average sodium intake reported in this cohort was also similar to the broader Australian cohort [43]. Furthermore, multiple outcome variables were able to be assessed in a single cohort as a result of the study population being well-characterised.

Limitations also need to be considered in the interpretation of these data. As a secondary analysis of data from a cross-sectional study, time-sensitive outcomes such as the age-related decline in taste [44] cannot be accounted for. As the cohort was 65 years and older, the results are not necessarily generalizable to the wider adult or youth population. However, the elderly cohort is suitable for the research question, as genetic and dietary exposures, and their interactions, accumulate over a lifetime and the relationships may only become apparent in older age. Dietary data collected with FFQs may be subject to reporting bias, including inaccurate recall, an under-reporting of quantities of discretionary food and over-reporting of healthful foods. Added salt used as seasoning is also likely to be under-reported via FFQs. However, the use of multiple dietary indices rather than a focus on estimated nutrient intakes improves the integrity of the body of data presented here. Future studies using 24-h urinary sodium should be conducted to confirm this result, as this would more precisely demonstrate intake. While the relationship between the *TRPV1*-rs8065080 genotype and the outcome variables are well-investigated in the present study, and limited associations found, interactions with other *TRPV1* SNPs or other ion channel taste receptor SNPs may exist. For this reason, polymorphic variant studies are needed in the future.

## 5. Conclusions

Additional studies are needed in more diverse age and cultural groups to determine if the lack of associations found between the *TRPV1*-rs8065080 genotype with sodium intake and markers of health is confined to the Caucasian elderly demographic. Furthermore, while this study was the first to characterise the relationship between *TRPV1*-rs8065080 genotype and markers of dietary intake, quality and health outcomes—data on perception, preference and sensitivity were not available, and further studies are needed to elucidate the differential impacts of oral versus extra-oral taste receptors. However, the extensive characterisation presented here will be important in the interpretation of the results of future studies assessing the relationship between the *TRPV1*-rs8065080 genotype, and dietary and health outcomes.

## Figures and Tables

**Table 1:** Distribution of continuous variables.

Variable	Mean	Minimum	Maximum	SD
Age (years)	77.4	65	94	6.8
DGI (150-point index)	97.0	30.9	132.6	15.8
ARFS (74-point index)	28.9	6	50	7.6
AUST-HEI (60-point index)	30.2	5	50	9.7
BMI (kg/m^2^)	28.5	17.1	46.3	4.8
Sodium intake (mg/day)	2052	506	8250	843

BMI: Body Mass Index.

**Table 2:** Distribution of categorical variables.

Variable	n	%
Sex		
Males	241	45.0
Females	295	55.0
Income		
<AUD 20,000 per year	165	31.5
AUD 20,000 to AUD 60,000 per year	322	61.6
>AUD 60,000 per year	36	6.9
Education		
≤Trade qualification	177	33.1
TAFE or other certificates	295	55.1
≥Bachelor degree	63	11.8
Smoking		
Current smoker	15	2.8
Ex-smoker	253	47.2
Never smoked	268	50.0
History of cardiovascular disease ^a,b^		
Yes	219	40.9
No	317	59.1
History of kidney disease ^b^		
Yes	90	17.5
No	425	82.5
Use of anti-hypertensive medication ^b^		
Yes	118	22.0
No	418	78.0
Regular use of any prescription medication ^b^		
Yes	438	81.7
No	98	18.3

^a^ includes hypertension, heart disease, stroke, heart attack and vascular disease; ^b^ self-reported.

**Table 3:** *TRPV1*-rs8065080 genotype distribution.

SNP	Female *n* (%)	Male *n* (%)	Total *n* (%)
C/C	33 (11.2)	33 (13.7)	66 (12.3)
C/T	134 (45.4)	116 (48.1)	250 (46.6)
T/T	128 (43.4)	92 (38.2)	220 (41.1)

**Table 4:** *TRPV1*-rs8065080 and urine creatinine and albumin to creatinine ratio.

	Unadjusted (Mean ± SEM)				Adjusted (Mean ± SEM)			
	C/C	C/T	T/T	p_trend_	C/C	C/T	T/T	p_trend_
Creat (mmol/L)	9.3 ± 0.6	9.7 ± 0.3	9.2 ± 0.3	0.5	9.1 ± 0.7	9.8 ± 0.4	9.2 ± 0.4	0.4
Alb/Creat Ratio (mg/mmol)	2.5 ± 4.0	9.3 ± 2.0	3.6 ± 2.3	0.1	5.7 ± 4.0	9.7 ± 2.3	7.5 ± 2.5	0.2

## Supplementary Materials

The following are available online at https://www.mdpi.com/2072-6643/12/4/1056/s1, Table S1: Stratified analyes, Table S2: Diet quality indices with sex, income, education and smoking.
