# Supplementary material for: Salt Taste Genotype, Dietary Habits and Biomarkers of Health: No Associations in an Elderly Cohort
Source: Nutrients. 2020 Apr 10;12(4):1056. doi: 10.3390/nu12041056 (PMC7231396; doi:10.3390/nu12041056)
Supplement: Supplementary file 1 [file nutrients-12-01056-s001.pdf]

Supplementary Table S1. Stratified Analyes

	Tukey HSD p-values												
	Sex		Age (years)		Income (per year)		Education			Smoking		BMI	
	Female	Male	65-79	≥80	<$20,000	>$20,000	≤Trade	TAFE/ certificate	≥Bachelor	Never smoked	History of smoking	≤24.9kg/m2	≥25kg/m2
**Sodium Intake (mg)**													
C/C - T/T	0.5	0.5	0.3	0.9	1.0	0.4	0.8	0.6	0.1	0.5	0.7	1.0	0.3
C/C - C/T	0.8	0.6	0.2	0.9	0.9	0.6	0.7	0.6	0.1	0.9	0.4	1.0	0.2
C/T - T/T	0.7	0.9	0.9	0.3	0.9	0.8	0.9	1.0	1.0	0.5	0.8	1.0	1.0
**DIET QUALITY INDICES**													
DGI													
C/C - T/T	0.3	0.9	0.9	0.3	1.0	0.5	0.6	0.4	0.4	0.7	0.5	0.3	0.6
C/C - C/T	0.6	0.9	0.7	1.0	0.9	0.8	0.8	0.6	0.9	0.9	0.7	1.0	0.4
C/T - T/T	0.7	1.0	0.8	0.1	1.0	0.9	0.8	0.8	0.6	1.0	0.9	0.1	0.9
ARFS													
C/C - T/T	0.2	0.9	0.9	0.2	1.0	0.7	0.7	0.4	0.8	1.0	0.4	0.2	0.6
C/C - C/T	0.6	0.9	0.8	0.9	1.0	0.4	0.9	0.7	1.0	1.0	0.7	0.9	0.5
C/T - T/T	0.5	1.0	1.0	0.1	1.0	0.7	0.9	0.7	0.8	1.0	0.7	0.1	1.0
AUST-HEI													
C/C - T/T	0.6	0.8	1.0	1.0	0.9	1.0	1.0	1.0	0.8	0.6	0.5	0.2	0.6
C/C - C/T	0.6	0.4	1.0	0.6	0.5	0.8	1.0	0.8	1.0	0.9	0.7	0.9	0.5
C/T - T/T	1.0	1.0	1.0	0.6	1.0	1.0	1.0	0.8	0.8	0.7	0.9	0.1	1.0
**URINE MARKERS**													
Creat (mmol/L)													
C/C - T/T	0.9	0.9	1.0	1.0	1.0	1.0	0.9	0.8	0.9	1.0	1.0	0.8	1.0
C/C - C/T	0.8	0.9	0.8	0.9	1.0	0.8	0.8	1.0	0.5	1.0	0.7	0.5	1.0
C/T - T/T	0.9	0.5	0.6	0.5	0.9	0.5	0.9	0.5	0.7	0.9	0.5	0.7	0.9
Alb (mmol/L)													
C/C - T/T	1.0	0.9	1.0	1.0	0.6	1.0	1.0	0.8	1.0	1.0	0.9	0.5	1.0
C/C - C/T	0.7	0.3	0.5	0.5	0.7	0.3	0.7	0.3	1.0	0.5	0.5	0.9	0.3
C/T - T/T	0.5	0.3	0.4	0.3	0.9	0.1	0.4	0.4	0.8	0.2	0.6	0.7	0.1
Alb/Creat Ratio (mg/mmol)													
C/C - T/T	1.0	1.0	1.0	1.0	1.0	1.0	1.0	0.8	1.0	1.0	0.9	0.6	1.0
C/C - C/T	0.7	0.4	0.6	0.5	0.6	0.5	0.7	0.3	1.0	0.5	0.6	0.9	0.3

C/T - T/T	0.5	0.3	0.4	0.4	0.4	0.4	0.4	0.4	0.9	0.2	0.6	0.6	0.1

Supplementary Table S2. Diet quality indices with sex, income, education and smoking

	DGI		AUST-HEI		ARFS	
**Variable**	**Mean ±SD**	**p**	**Mean ±SD**	**p**	**Mean ±SD**	**p**
Sex						
Males	95.6±15.0	**0.007**	28.7±9.7	**<0.0001**	27.0±7.7	0.1
Females	99.7±16.1		32.6±9.0		28.3±7.8	
Income						
< $20,000 per year	97.9±16.8	0.8	30.7±9.8	0.9	27.9±8.3	0.7
>$20,000 per year	97.4±15.1		30.7±9.3		27.5±7.5	
Education						
≤ Trade qualification	95.4±16.5		29.1±9.7		26.7±7.8	
TAFE or other certificates	97.2±15.6	**0.04**	31.0±9.6	**0.02**	27.8±7.8	0.8
≥ Bachelor degree	100.3±14.8		32.1±8.6		28.6±7.0	
Smoking						
History of smoking	96.1±15.6	**0.03**	30.5±9.6	0.7	27.1±7.6	0.1
Never smoked	99.2±15.8		20.8±9.3		28.3±7.9	
